# Deregulation of TLR4 signaling pathway characterizes Bicuspid Aortic valve syndrome

**DOI:** 10.1038/s41598-019-47412-0

**Published:** 2019-07-30

**Authors:** Carmela R. Balistreri, Antonino G. M. Marullo, Michele Madonna, Elena Cavarretta, Alberto Allegra, Valeriana Cesarini, Alessandra Iaccarino, Sonia Schiavon, Mariangela Peruzzi, Ernesto Greco, Sebastiano Sciarretta, Calogera Pisano, Giovanni Ruvolo, Michele Torella, Giacomo Frati

**Affiliations:** 1 Department of Biomedicine, Neuroscience and Advanced Diagnostics (Bi.N.D.), University of Palermo, Palermo, Italy; 2 Department of Medico-Surgical Sciences and Biotechnologies, Sapienza University of Rome, Latina, Italy; 3 IRCCS Neuromed, Pozzilli, IS Italy; 4 Mediterranea Cardiocentro, Naples, Italy; 5 Department of Surgery and Oncology, University of Palermo, Palermo, Italy; 6 Department of Biomedicine and Prevention, Tor Vergata University, Rome, Italy; 7 Department of Cardiac Surgery, Humanitas Clinical and Research Center, Rozzano, Italy; 8 Department of Cardiovascular, Respiratory, Nephrological, Anesthesiological, and Geriatric Sciences, Sapienza University of Rome, Rome, Italy; 9 Department of Cardiac Surgery, University of Rome “Tor Vergata,”, Rome, Italy; 10 Department of Cardiothoracic Sciences, University of Campania “L. Vanvitelli”, Naples, Italy

**Keywords:** Cardiology, Valvular disease

## Abstract

Bicuspid aortic valve (BAV) disease is recognized to be a *syndrome* with a complex and multifaceted pathophysiology. Its progression is modulated by diverse evolutionary conserved pathways, such as Notch-1 pathway. Emerging evidence is also highlighting the key role of TLR4 signaling pathway in the aortic valve pathologies and their related complications, such as sporadic ascending aorta aneurysms (AAA). Consistent with these observations, we aimed to evaluate the role of TLR4 pathway in both BAV disease and its common complication, such as AAA. To this aim, 70 subjects with BAV (M/F 50/20; mean age: 58.8 ± 14.8 years) and 70 subjects with tricuspid aortic valve (TAV) (M/F 35/35; mean age: 69.1 ± 12.8 years), with and without AAA were enrolled. Plasma assessment, tissue and gene expression evaluations were performed. Consistent with data obtained in the previous study on immune clonotypic T and B altered responses, we found reduced levels of systemic TNF-α, IL-1, IL-6, IL-17 cytokines in BAV cases, either in the presence or absence of AAA, than TAV cases (p < 0.0001 by ANOVA test). Interestingly, we also detected reduced levels of s-TLR4 in BAV cases with or without AAA in comparison to the two groups of TAV subjects (p < 0.0001 by ANOVA test). These results may suggest a deregulation in the activity or in the expression of TLR4 signaling pathway in all BAV cases. Portrait of these data is, indeed, the significantly decreased gene expression of inflammatory cytokines and TLR4, in both normal and aneurysmatic tissue samples, from BAV with AAA than TAV with AAA. In conclusion, our study demonstrates that subjects with BAV display a significant deregulation of TLR4 signaling pathway paralleled by a deregulation of Notch-1 pathway, as previously showed. This data suggests that the crosstalk between the Notch-1 and TLR4 signaling pathways may play a crucial role in both physiological embryological development, and homeostasis and functionality of aortic valve in adult life.

## Introduction

A novel evidence sustains that the Toll-like receptor-4 (TLR4) signaling pathway has the role of hub in preserving aorta homeostasis, but also in contributing to the onset of degenerative aorta diseases, such as aneurysms^1^. Consistent with this, it is emerging the key role of TLR4 signaling pathway in the complex pathophysiology of sporadic ascending aortic aneurysms (AAA)^1^. Accordingly, we recently postulated *the model of the signaling pathway from the double*-*face*^1^, supported by results of the recent investigations performed by our and other groups^2–9^. Specifically, it proposes that AAA is the result of a sustained/excessive activation of TLR4 signaling pathway, expressed on both endothelial and vascular smooth muscle cells, followed by its cross-talk with other pathways, including the Notch pathway, but also by TGF-β, NO, MMP, NF-Kβ pathways^1,8^.

Here, we investigated if TLR4 signaling pathway is involved as one of the main determinants of both pathophysiology and early onset of AAA in patients affected by bicuspid aortic valve (BAV). BAV is the most common congenital cardiac malformation, affecting approximately 1.3% of the population worldwide^10–12^ with AAA and dissection representing its common complications, even if the related mechanisms are not completely known^10–12^. We recently evidenced the presence of distinctive molecular and cellular mechanisms in BAV patients, when compared to tricuspid aortic valve (TAV) patients with or without AAA^11–13^. Specifically, we demonstrated an increased allele frequency of some polymorphisms in genes encoding molecules of NO, MMP, ACE pathways^13^, a quantitative reduction in the circulating levels of T and B lymphocytes cell subsets^14^, a deregulation of Notch 1 pathway and circulating endothelial progenitor cell (EPC)^15^ number in BAV cases compared to TAV cases, irrespective from the presence of AAA disease.

TLR4 signaling pathway is known to modulate both the expression and function of the above-mentioned pathways^1^. A close crosstalk between TLR-4 and the abovementioned pathways has been proposed^1,16^, which seems to explain the multitude of TLR4 actions and its biological effects on the cardiovascular system, as well as in the aorta and aortic valve. Of crucial importance, is the crosstalk between TLR-4 and Notch pathways^1,16^. A growing evidence supports these observations, as reported in previous works^1,16^.

Thus, alterations in these pathways result in a deregulation of vascular homeostasis and valvulogenesis, followed by both valve and vascular structure abnormalities and the onset of pathological conditions, such as valve degeneration, endothelial dysfunction, medial degeneration and vascular degeneration, significantly associated with the development and progression of AAA. Accordingly, we hypothesized that a deregulation of TLR-4 pathway could be related to BAV disease, thereby contributing to AAA onset and progression. Consequently, the main aim was to evaluate TLR-4 signaling in a cohort of BAV and TAV patients, with or without AAA^13^.

## Results

### Systemic levels of TNF-α, IL-6, IL-1, IL-17 and s-TLR4

First, we tested blood levels of TNF-α, IL-1, IL-6 and IL-17 in plasma samples, since accumulating lines of evidence pointed out a crucial role of inflammation of the aortic wall in contributing to the development and progression of aortic aneurysm^1,8,9,11,17–19^. The comparisons, among the four groups, demonstrated that the systemic levels of these cytokines were lower in BAV subjects without AAA with respect to other groups (see Table 1). In BAV subjects with AAA, we observed a significant increase of systemic levels of these cytokines when compared to the levels of BAV cases without AAA. In contrast, TAV individuals showed higher significant levels of these cytokines independently of the presence or absence of AAA, when compared to BAV cases with or without AAA. The presence of AAA determined a rise of inflammatory cytokines in BAV cases, but to a lesser extent than TAV individuals (see Table 1). Thus, a deregulated immune response seems to occur in BAV cases.

**Table 1:** Systemic levels of TNF-α, IL-1, IL-6, IL-17, and s-TLR4.

Systemic molecules examined	BAV without AAA N = 19	BAV with AAA N = 51	P*
TNF-α (pg/ml)	8.18 ± 1.9	28 ± 5.4	**<0.0001**
IL-6 (pg/ml)	20 ± 4.3	68 ± 5.6	**<0.0001**
IL-1 (pg/ml)	22 ± 1.4	55 ± 8	**<0.0001**
IL-17 (pg/ml)	16 ± 1.9	78 ± 6	**<0.0001**
s-TLR4 (ng/ml)	1.5 ± 1.8	8.5 ± 2.1	**<0.0001**
**Systemic molecules examined**	**TAV without AAA N** = **45**	**TAV with AAA N** = **25**	**P**
TNF-α (pg/ml)	12.7 ± 4.3	75 ± 5.3	**<0.0001**
IL-6 (pg/ml)	28 ± 9	90 ± 8	**<0.0001**
IL-1 (pg/ml)	29 ± 6.5	72.4 ± 6	**<0.0001**
IL-17 (pg/ml)	21 ± 1.9	98 ± 6	**<0.0001**
s-TLR4(ng/ml)	4.5 ± 1.5	18 ± 4	**<0.0001**
**Systemic molecules examined**	**BAV without AAA N** = **19**	**TAV without AAA N** = **45**	**P**
TNF-α (pg/ml)	8.18 ± 1.9	12.7 ± 4.3	**0.01**
IL-6 (pg/ml)	20 ± 4.3	28 ± 9	**0.01**
IL-1 (pg/ml)	22 ± 1.4	29 ± 6.5	**0.01**
IL-17 (pg/ml)	16 ± 1.9	21 ± 1.9	**0.01**
s-TLR4 (ng/ml)	1.5 ± 1.8	4.5 ± 1.5	**0.01**
**Systemic molecules examined**	**BAV with AAA N** = **51**	**TAV with AAA N** = **25**	**P**
TNF-α (pg/ml)	28 ± 5.4	75 ± 5.3	**0.0001**
IL-6 (pg/ml)	68 ± 5.6	90 ± 8	**0.001**
IL-1 (pg/ml)	55 ± 8	72.4 ± 6	**0.001**
IL-17 (pg/ml)	78 ± 6	98 ± 6	**0.001**
s-TLR4(ng/ml)	8.5 ± 2.1	18 ± 4	**0.001**

Tumor necrosis factor (TNF)-α, Interleukin (IL)-1, IL-6, IL-17; soluble-Toll-like receptor (sTLR)4.

*By unpaired t-test with Welch correction.

We also assessed the systemic levels of soluble TLR4 (s-TLR4) in plasma samples of the four groups enrolled. Levels of s-TLR4 depict the presence or absence of a constitutive balance between activation and inhibition of TLR4 signaling pathway^1,20–22^, representing decoy receptors with inhibitor action. This allows the control of TLR4 physiological actions in the *milieu* of the aortic wall, that is important for the homeostasis of aortic tissue^1^. Interestingly, we found reduced levels of s-TLR4 in BAV cases with or without AAA in comparison to the two groups of TAV subjects (1.5 ± 1.8 and 8.5 ± 2.1 vs. 12.7 ± 4.3 and 75 ± 5.3(ng/ml), respectively). This data should seem to indicate a deregulation in the activity or in the expression of TLR4 signaling pathway in all BAV cases.

### Expression levels of TLR4, IL-1β, IL-6, and IL-17 genes in aortic tissues from BAV and TAV patients

**Figure 1:**
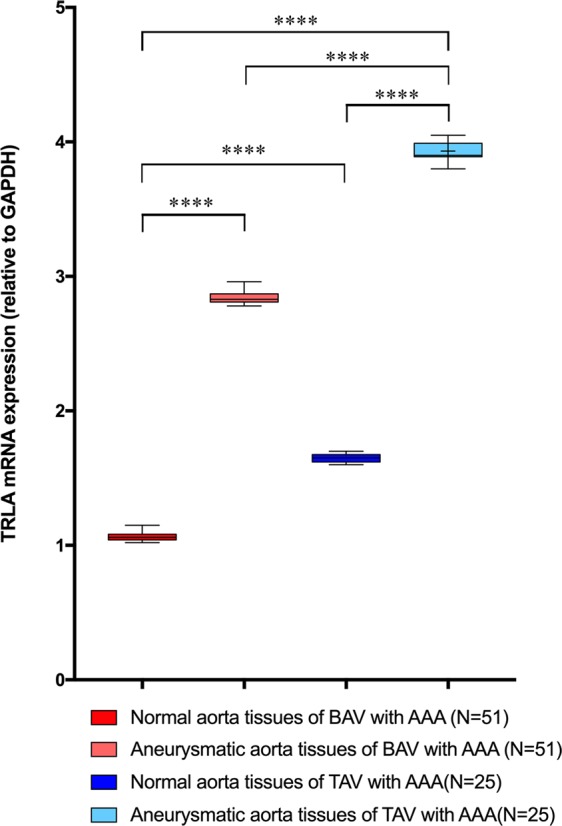
Expression levels of gene encoding TLR4 (relative to GAPDH). The figure reports the gene expression levels of TLR4 in normal or aneurysmatic tissue samples from 51 BAV and 25 TAV cases with AAA. The comparison of data showed significant differences among the four groups with p < 0.0001, by ANOVA test, Bonferroni corrected. Significant also were the levels of TLR4 gene expression by comparing data in normal aorta tissues from BAV and TAV cases (p = 0.0001, by t test, Welch corrected), and between two levels in aneurymatic tissue samples from TAV and BAV groups with AAA (p = 0.0001, by t test, Welch corrected). However, the lowest levels were observed in normal aorta tissues from BAV with AAA, that confirmed their systemic levels of s-TLR4. The statistical significance was detected by using t test and ANOVA test, when appropriate.

To validate the significant reduction of plasma levels of inflammatory cytokines and the soluble form of TLR4 in all BAV subjects, we also evaluated the expression levels of TLR4 and IL-1β, IL-6, IL-17 genes in aorta tissues harvested from patients with BAV and TAV affected by AAA undergoing surgery. Specifically, they were detected in normal (control samples) and aneurysm aortic portions harvested from patients undergoing elective surgical repair. As represented in Fig. 1, the comparison of the levels of TLR4 gene expression, among the four groups, revealed significant differences (p < 0.0001, by ANOVA test, Bonferroni corrected, or t test, Welch corrected).

**Figure 2:**
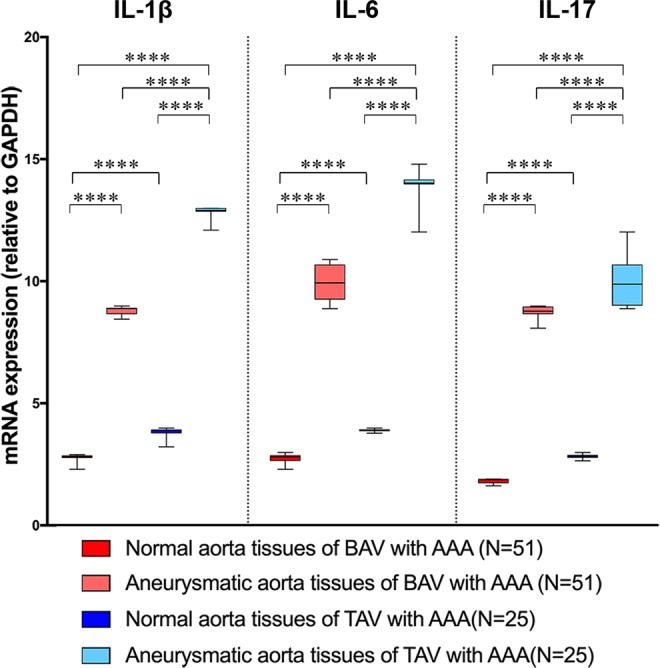
Expression levels of genes encoding inflammatory cytokines. The figure reports the gene expression levels of IL-1β, IL-6 and IL-7 in normal or aneurysmatic tissue samples from 51 BAV and 25 TAV cases with AAA. The comparison of data of each cytokine gene detected significant differences among the four groups with p < 0.0001, by ANOVA test, Bonferroni corrected, and between normal and aneurysmatic aorta tissues from BAV and TAV cases (p = 0.0001, by t test, Welch corrected), with the lowest peaks in all the aorta tissues from BAV cases. The statistical significance was detected by using t test and ANOVA test, when appropriate.

Of note, we observed that the levels of TLR4 gene expression were significantly lower in both, normal and aneurysmatic aorta samples from BAV cases with or without AAA in comparison to TAV cases with or without AAA (see Fig. 1). They increased in BAV aneurysmatic aorta samples, but they showed, significant reduced values, when compared to gene expression levels of TLR4 in aneurysmatic aorta tissue samples from TAV cases with AAA (see Fig. 1). Likewise, we detected similar data by comparing the expression levels of IL-1β, IL-6, IL-17 genes in normal aorta and aortic aneurysmatic samples from BAV and TAV patients (see Fig. 2). Increased gene expression was observed in aneurysmatic samples from both BAV and TAV patients, with higher levels observed in TAV cases (p = 0.0001, by t test, Welch corrected). Of note, a lowest expression of these four (TLR4, IL-1β, IL-6, IL-17) genes was detected in in normal aortic tissue samples from BAV cases (see Figs. 1, 2).

## Discussion

Chronic inflammation is recognized to be involved in degenerative pathological conditions of both aorta and aortic valve^1,8,9,17–19^. The early degeneration of aortic valve, accompanied by several serious complications (i.e. AAA, the most common), usually occurs in case of bicuspid valve condition^10–12^. BAV, initially considered the result of a simple embryonic defect during the valvulogenesis, is today recognized to have a complex pathophysiology^11,12,23^ and progression mediated by diverse evolutionary conserved pathways, such as Notch-1, as recently demonstrated by our group^10,11,15^. Established evidence is also demonstrating the key role of inflammatory pathways, such as TLR4 signaling pathway, in the aortic valve pathologies, such as calcific aortic valve disease^11,15^. In aortic valve, TLR4 signaling pathway is expressed in both human aortic valve endothelial and interstitial cells^24–27^, and mediates its biological effects fundamentally via modulation of Notch-1 pathway^28^. Recently, we demonstrated and emphasized this concept and the crucial role of TLR-4 signaling pathway in the onset and progression of AAA^1,8,15,16^.

Consistent with these observations, in this study we aimed to evaluate the role of this pathway in BAV disease and its common complication, such as AAA. We found reduced levels of both gene and tissue expression of TLR4 signaling pathway in BAV cases, independently of the presence or absence of AAA. Portrait of these data is the decreased gene expression of inflammatory cytokines, that we detected in aorta tissue samples from BAV cases in comparison to TAV cases. We also observed significant reduced systemic levels of these cytokines in plasma samples from BAV cases, and particularly in those without AAA.

Thus, a deregulated gene ad tissue expression of TLR4 signaling pathway seems to characterize BAV disease. This signature might also explain the reduced levels of the regulatory molecules, such as s-TLR4, detected in plasma samples from BAV cases in respect to TAV subjects. In addition, the concomitant deregulation of Notch-1 pathways, previously observed in BAV cases^15^, might also justify the reduced numbers of circulating T and B subsets and EPC^14,15^. These two pathways are, indeed, known to play a crucial role in physiological haematopoiesis^29,30^, the physiological release and functionality of progenitors, such as EPC^8,30,31^, as well as in embryological organogenesis and valvulogenesis^1,16,23,30,32^.

For these reasons we support the concept of BAV as a syndrome^11,12,23^, with a complex pathophysiology, characterized by the deregulation of TLR4 and Notch-1 pathways, that are known to be crucial for the physiological homeostasis and function of aortic valve endothelial (AVEC) and interstitial (AVIC) cells, as well as the aorta wall cells. Consequently, we propose that the altered function and expression of these two pathways, observed in BAV disease, result in a reduced functionality of AVEC, AVIC and aortic wall cells, with an increased susceptibility to senescence and chronic inflammation finally leading to a functional impairment^8,9,16,33–35^.

### Study limitations

The principal limitation of the study relies on the fact that it is associative, and this could limit the identification of the true cause-effect relationship between TLR4 signaling pathway and BAV disease, and its complication AAA. Regarding this aspect, it should also consider that the TLR-4 function and expression are modulated not only by genetic variants and haplotypes^1,9,36^, but also by environmental factors (such as diet^37^, mite allergens and air pollution^38^), and their cross-interaction with microbiota^37^, which may remain in a healthy state or show alterations (i.e. dysbiosis and consequent endotoxemia associated with age or obesity)^39^ and consequent epigenetic changes^40^. Current evidence is also demonstrating that  pharmacological treatments, such as antihypertension drugs (i.e. candesartan, an angiotensin type 1 receptor (AT1R) blocker) can inhibit the TLR4/Angiotensin II-induced NF-κB inflammatory signaling^41^. Thus, differences in the pharmacological therapy administrated to various patients may represent another confounding factor. However, in our study, all the BAV and TAV patients were subjected to the same antihypertensive treatment. Furthermore, no investigations in this study were also performed for evaluating, whereas the reduced expression and function of TLR4 pathway correlated with a reduced grade of renewal and senescence of AVEC components of BAV valve than TAV valve. This might confirm the important data obtained in the previous study about the reduced circulating levels of EPC detected in BAV cases independently by the presence or not of an associated aortopathy. Lastly, another limitation might be represented by the fact that age was lower in subjects with BAV thereby hypothetically interfering with the results even if this depends on the early onset of complications in BAV cases with respect to TAV cases.

## Conclusions

In conclusion, our study demonstrates that subjects with BAV display a significant deregulation of TLR4 signaling pathway accompanied by a deregulation of Notch-1 pathway, as previously showed^15^. This data suggests that the crosstalk between the Notch-1 and TLR4 signaling pathways may play a crucial role in both physiological embryological development, and homeostasis and functionality of aortic valve in adult life. Further investigations by means of new technologies, such as Cre/Lox recombination, might help to clear this aspect, as recently reported in literature^42^. The validation and confirmation of the relevance of these pathways may lead to use them as potential therapeutic targets for personalized treatments, until now inexistent for both BAV and AAA, and to identify a potentially suitable biomarker profile useful to facilitate management and outcome of this complex syndrome. Certainly, additional and larger studies, and the use of methodologies and technologies of new generation, such as the abovementioned Cre/Lox recombination^42^, are mandatory to validate and confirm these promising findings, as well as our suggestions.

In this context, investigations that clarify the effects of environmental triggers, such as diet, allergens, air pollution, drugs in the pathophysiology of BAV and AAA complication through activation of TLR-4 pathway, including the intriguing link between the intestinal microbiota and BAV mediated by the TLR4-pathway^43^, might also be helpful. Since everyone is the result of the sophisticated interplay between environmental factors and its genome, trascriptome, proteome, metabolome, microbiome, epigenome, exposome, we here suggest that it is necessary to perform a more complex combination of investigations based on these aspects for obtaining interesting data in the study of BAV and AAA complication^1,11,12^.

## Material and Methods

### Study population

The population of 70 BAV subjects (50 males and 20 females; mean age: 58.8 ± 14.8 years) and 70 TAV subjects (35 males and 35 females; mean age: 69.1 ± 12.8 years) with or without AAA, previously enrolled in the recent study from Balistreri and coworkers^15^, was also included in this investigation. Briefly, it was recruited from January 2015 to December 2016. The cases were randomly selected from patients referring to the Units of Cardiac Surgery (Department of Surgery and Oncology, University of Palermo) and Cardiology, for surgery replacement or routine care screening. Appropriate exclusion criteria were also used during the BAV/TAV enrollment, for the following diseases: a) cardiovascular diseases were excluded according to history and by detecting apposite laboratory and imaging biomarkers as indicated by more recent ESC or ASC guidelines; b) connective tissue disorders were excluded by assessing markers of inflammation immunological (i.e. autoantibodies) and imaging biomarkers; c) inflammatory diseases (from infections to hematological, gastrointestinal, urogenital, pulmonary, neurological, endocrinal inflammatory disorders, and neoplasies included) by detecting apposite laboratory (including complete blood cell count, erythrocyte sedimentation rate, glucose, urea nitrogen, creatinine, electrolytes, C reactive protein, liver function tests, iron, and proteins) and imaging biomarkers. In addition, all the cases enrolled belonged to the same ethnic group in Western Sicily. Thus, a very homogenous population was studied. Furthermore, elective or acute surgical treatment (using wheat operation, Bentall-De Bono and Tirone David surgical techniques, whenever possible) and complementary tubular-ascending aorta resection were performed in the BAV and TAV patients with AAA after evaluation of aortic transverse diameter sizes by Computed Tomography scanning according to recent guidelines according to recent guidelines, as reported in our recent review^9^. Accordingly, an experienced physician evaluated aortic transverse diameter sizes by echocardiography (Philips Ie. 33) before either elective or urgent surgery. The dimension of the aortic annulus, sinuses of Valsalva, proximal ascending aorta (above 2.5 cm of the sino-tubular junction) and aortic arch were assessed pre-operatively by trans-thoracic echocardiography as well as in the operating theatre by trans-oesophageal-echocardiography before the institution of the cardiopulmonary by-pass. These measures, together with demographic and clinical data (including comorbidities) were obtained from patients’ medical records and are presented in Table 2. In all BAV and TAV cases, hypertension was treated by beta-blockers.

**Table 2:** Demographic and clinical characteristics, comorbodity conditions, complications of 70 BAV and 70 TAV subjects.

Variables		BAV N = 70	TAV N = 70	P
*Demographic characteristics*	Age, mean (SD)	58.8 (14.8)	69.1(12.8)	<0.0001
	Male sex, No. (%)*	50 (71)	35(50)	0.009
	Female sex, No. (%)	20 (29)	35 (50)	0.009
	Body mass index, mean (SD)	26 (4.8)	26.3 (3.2)	N.S.
*Size and location of AAA*	Subjects affected (%)	51 (73)	25(36)	0.00001
	Size (mm), mean (SD)	53.3 (7.4)	50.3 (6.9)	N.S.
	Location, No:			
	Tubular ascending aorta	51 (100)	25 (100)	N.S.
*Comorbidity conditions*, *No (%)*	CVD Family History	8 (11)	5 (7.1)	N.S.
	Smoking	26 (37)	20 (28)	N.S.
	Hypertension	55 (78)	50 (719	N.S.
	Dyslipidemia	9 (13)	5 (7.1)	N.S.
	Diabetes mellitus	3 (4.2)	1 (1.4)	N.S.
	Renal failure	0 (0)	1 (1.4)	N.S.
	Dissection	0 (0)	0 (0)	N.S.
*Aortic valve pathology*, *No (%):*				
	Normal	0 (0)	38 (54)	0.0001
	Prolapse	8 (11)	0(0)	0.003
	Vascular calcium fibrosis	35 (50)	7 (10)	2.4e-7
*Atherosclerosis coronary syndrome*, *No (%):*		2 (3)	1 (0.8)	N.S.

*Percentage values on total BAV and TAV subjects.

P = TAV vs. BAV, by t Student test for quantitative variables, or *χ*^2^ test for qualitative variables.

### Ethical Study Approval

Our study was performed in accordance with ethical standards of the Helsinki Declaration of the World Medical Association and Italian legislation, and it received approval from Regional Ethics Board in Palermo (No. APUNIP0094517)^13^ and all participants gave their informed consent. Data were encrypted to ensure patient s’ privacy. All clinical measurements were performed in blind.

### Quantifications of systemic levels of TNF-α, IL-6, IL-1, IL-17 and sTLR4

Venous blood samples were collected from all enrolled subjects in a fasting state (>8 h without food administration). In BAV and TAV cases, blood samples were collected within the first 3 h of their admission. Plasma samples were obtained after a centrifugation of 3500 rpm at 4 °C for 10 min immediately after collection and then stored at −80 °C for further analysis. Plasma TNF-α, IL-6, IL-1 IL-17 levels were measured by using ELISA technique and commercial kits (R&D Systems, Minneapolis, MN, USA), according to the manufacturer’s instructions. Detection limits were 0.7 pg/ml, 0.5 pg/ml, 1 pg/ml, 15 pg/ml for IL-6, TNF-α, IL-1, IL-17 respectively. All assays were run in duplicate. Regarding the quantification of blood sTLR4 concentrations, they were also measured by a commercial ELISA kits (USCN Life Science, Inc., Wuhan, China) with a lower limit of detection of 0.156 ng/ml.

### Transcription analyses by using Real-time PCR (qRT-PCR)

Full aortic segments with normal as well as aneurysmatic aortic wall from tubular-ascending aorta were collected from patients with AAA. Specimens were fixed in 10% neutral buffered formalin and then processed for routine paraffin embedding. Total RNA was extracted by using the Qiagen RNeasy FFPE kit, treated with DNase I enzyme (Promega) for 1 h at 37 °C and then cleaned by column purification (Qiagen). Total RNA concentration and quality were determined with a spectrophotometer. Then cDNA was prepared using 1–2 µg of RNA (Ready-To-Go, T-Primed First-Strand Kit, Amersham Bioscience). The synthesised cDNA was stored at -20 °C RT-PCR analysis. They were also successively utilized for evaluating the TLR4, IL-1β, IL-6 and IL-17 mRNA expression. In these analyses, we used the expression of a house-keeping gene, the glyceraldehyde-3-phosphate dehydrogenase (GAPDH), as endogenous control for normalization of the amount of sample RNA. The reactions were also performed using SYBR Green (catalog no. QR0100 Sigma-Aldrich) qRT-PCR in a Light-Cycler (Roche). The sequences of TLR4, cytokines and GAPDH primers used were reported in Table 3. The results were analysed using the 2^−ΔCt^ (Livak) relative expression method.

**Table 3:** Primer sequences for qRT-PCR.

Human gene	Forward	Reverse
TLR-4	5′-AATCTAGAGCACTTGGACCTTTCC -3′	5′-GGGTTCAGGGACAGGTCTAAAGA -3′
IL-1 β	5′-CTGTCCTGCGTGTTGAAAGA-3′	5′-TTGGGTAATTTTTGGGATCTACA-3′
IL-6	5′-TCTCCACAAGCGCCTTCG-3′	5′-CTCAGGGCTGAGATGCCG-3′
IL-17	5′-CTCATTGGTGTCACTGCTACTG-3′	5′ CCTGGATTTCGTGGGATTGTG-3′
GAPDH	5′-GGTATCGTCGAAGGACTCATGAC-3′	5′-ATGCCAGTGAGCTTCCCGTTCAGC-3′

### Statistical analysis

Statistical analyses were performed using SPSS software version 20. Significant differences among qualitative variables were calculated by using *χ*^2^ test. Continuous variables (including systemic blood molecule, protein and gene expression levels) were expressed as mean ± SD (Standard deviation). Unpaired *t*-test (Welch corrected) was utilised to analyse the data between two groups, while one-way ANOVA or Kruskal-Wallis test followed by Bonferroni correction was applied to compare more than two groups. To identify possible correlations, a non-parametrical Spearman correlation test was also used. Differences were considered significant when a p value < 0.05 was obtained by comparison between the different groups.
